# Phylogenetic debugging of a complete human biosynthetic pathway transplanted into yeast

**DOI:** 10.1093/nar/gkz1098

**Published:** 2019-11-20

**Authors:** Neta Agmon, Jasmine Temple, Zuojian Tang, Tobias Schraink, Maayan Baron, Jun Chen, Paolo Mita, James A Martin, Benjamin P Tu, Itai Yanai, David Fenyö, Jef D Boeke

**Affiliations:** 1 Institute for Systems Genetics and Department of Biochemistry and Molecular Pharmacology, NYU Langone Health, New York, NY, USA; 2 Memorial Sloan Kettering Cancer Center, New York, NY, USA; 3 Center for Genomics and Systems Biology, Department of Biology, New York University, New York, NY, USA; 4 Institute for Computational Medicine and Department of Biochemistry and Molecular Pharmacology, NYU Langone Health, New York, NY, USA; 5 Department of Biochemistry, The University of Texas Southwestern Medical Center, Dallas, TX, USA

## Abstract

Cross-species pathway transplantation enables insight into a biological process not possible through traditional approaches. We replaced the enzymes catalyzing the entire *Saccharomyces cerevisiae* adenine *de novo* biosynthesis pathway with the human pathway. While the ‘humanized’ yeast grew in the absence of adenine, it did so poorly. Dissection of the phenotype revealed that *PPAT*, the human ortholog of *ADE4*, showed only partial function whereas all other genes complemented fully. Suppressor analysis revealed other pathways that play a role in adenine *de-novo* pathway regulation. Phylogenetic analysis pointed to adaptations of enzyme regulation to endogenous metabolite level ‘setpoints’ in diverse organisms. Using DNA shuffling, we isolated specific amino acids combinations that stabilize the human protein in yeast. Thus, using adenine *de novo* biosynthesis as a proof of concept, we suggest that the engineering methods used in this study as well as the debugging strategies can be utilized to transplant metabolic pathway from any origin into yeast.

## INTRODUCTION

Classical genetics and biochemistry approaches have been used to study genetic and metabolic networks for decades. The surge in the availability of genomic information and molecular tools over the past three decades has opened new opportunities to reveal how these networks function and can be manipulated. With the genomes of >60 000 organisms sequenced (NCBI) and access to millions of human variants (e.g. 1000 genomes, GWAS), we can now reveal regulatory mechanisms by combining classical approaches with the technologies that are driving the fast-growing emerging field of synthetic biology.

Genome sequencing has exposed us to a large pool of variants that adapted to different cellular and natural environments. However, the ability to study the functionality of a regulatory or coding segment of DNA remains the largest bottleneck to understanding it. Yeast and bacteria have been extensively used as model organisms to study complex cellular processes. As a eukaryotic hosts, the characteristics of the yeast cellular network is presumably much more compatible for characterization of heterologous eukaryotic pathways and genes. Years of genetic manipulation have created a large toolkit of molecular biology tools that can be used to assemble and express foreign DNA in yeast.

Single human genes have been transplanted into yeast for decades (1–5). Modern cloning and screening tools have allowed scientists to substantially increase the scale with which they can engineer genes into model organisms (6–8). Recent advances in synthesis technology, driven by the synthetic biology field, made it possible to synthesize bigger and more complex DNA molecules. Advances in molecular engineering have enabled us to synthesize the DNA that encodes entire pathways. Despite several attempts at multigene transplantation across species boundaries (9–11), transplantation of a full functional human pathway into yeast has been elusive.

Despite enormous variation in adaptation to different/similar environments between species in the tree of life, basic metabolic tasks are highly conserved (12). However, all organisms are auxotrophic and need to scavenge for nutrients from their environment which can differ vastly among diverse species. Thus, it is easy to assume that even highly conserved fundamental metabolic tasks have evolved to accommodate the needs of a cell in its specific milieu, creating a cell/organism-specific metabolic ‘set-point’, but - is there actual evidence for this? One possible way to answer this question would be comparative metabolomics between different organisms however, an extensive quantitative study has not yet been done. Nevertheless, there are a few pieces of evidence that might suggest the existence of such differences. Any phylogenetic comparison of sequences shows differences between orthologues from different species. Although some of these variations are neutral and occur randomly, some may reflect the organism's adaptation to its environment (13). *In vitro* data shows that orthologous proteins have quite distinct biochemical properties (14), suggesting that they evolved to operate under distinct conditions and to function optimally with different concentrations of metabolites.

Here, we report an unbiased approach to transplant into yeast, in a single shot, multiple human enzymes that are part of a metabolic network. We show, for the first time, the transplantation of 7 human genes, constituting the adenine *de novo* pathway, into yeast cells. We expose *ADE4/PPAT* as the key regulatory node of the pathway and, using phylogenetic analysis of *PPATs* from 70 different organisms, isolate the key residues involved in Ppat regulation. This defines a new strategy for pathway engineering informed by evolutionary differences analyzed by coupling cross-species transplantation with auxotrophic complementation using phylogenetically distinct orthologs. In addition, we provided *in vivo* evidence for the adaptation of metabolic enzymes and their regulation to their cellular environment.

## MATERIALS AND METHODS

### Strains and media

Yeast strains and the plasmids contained are listed in Supplementary Table S4. All strains are derived from BY4741 (*MAT****a****leu2Δ0 met15Δ0 ura3Δ0 his3Δ1*) and BY4742 (*MATα leu2Δ0 lys2Δ0 ura3Δ0 his3Δ1*) (15). Media used were as follows. SD-based media supplemented with appropriate amino acids; fully supplemented medium containing all amino acids plus uracil and adenine is referred to as SC (16,17). Throughout this report we refer to medium as SC—a nutrient, indicating SC medium lacking the appropriate supplement(s) necessary to maintain specific constructs in the strains. Thus the medium either contains adenine (1.6 mM final concentration) or lacks it (in SC–Ade). In addition, when cells were grown for prolonged periods of time in SC liquid medium, adenine was supplemented at 8mM final concentration, this is referred to as SCA. β-Estradiol was purchased from Sigma-Aldrich (St Louis, MO, USA), and 5-fluoroorotic acid (5-FOA) was from US Biological (Massachusetts, MA, USA). Yeast strains were also cultured in YEPD medium (16,17) or YEPD supplemented with 200 μg/ml Geneticin (G418) sulfate (Santa Cruz Biotechnology, sc-29065B).

*Escherichia coli* was grown in Luria Broth (LB) media. To select strains with drug-resistant genes, carbenicillin (Sigma-Aldrich) or kanamycin (Sigma-Aldrich) were used at final concentrations of 75 and 50 μg/ml respectively. Agar was added to 2% for preparing solid media.

### Plasmids

**Figure 1.**
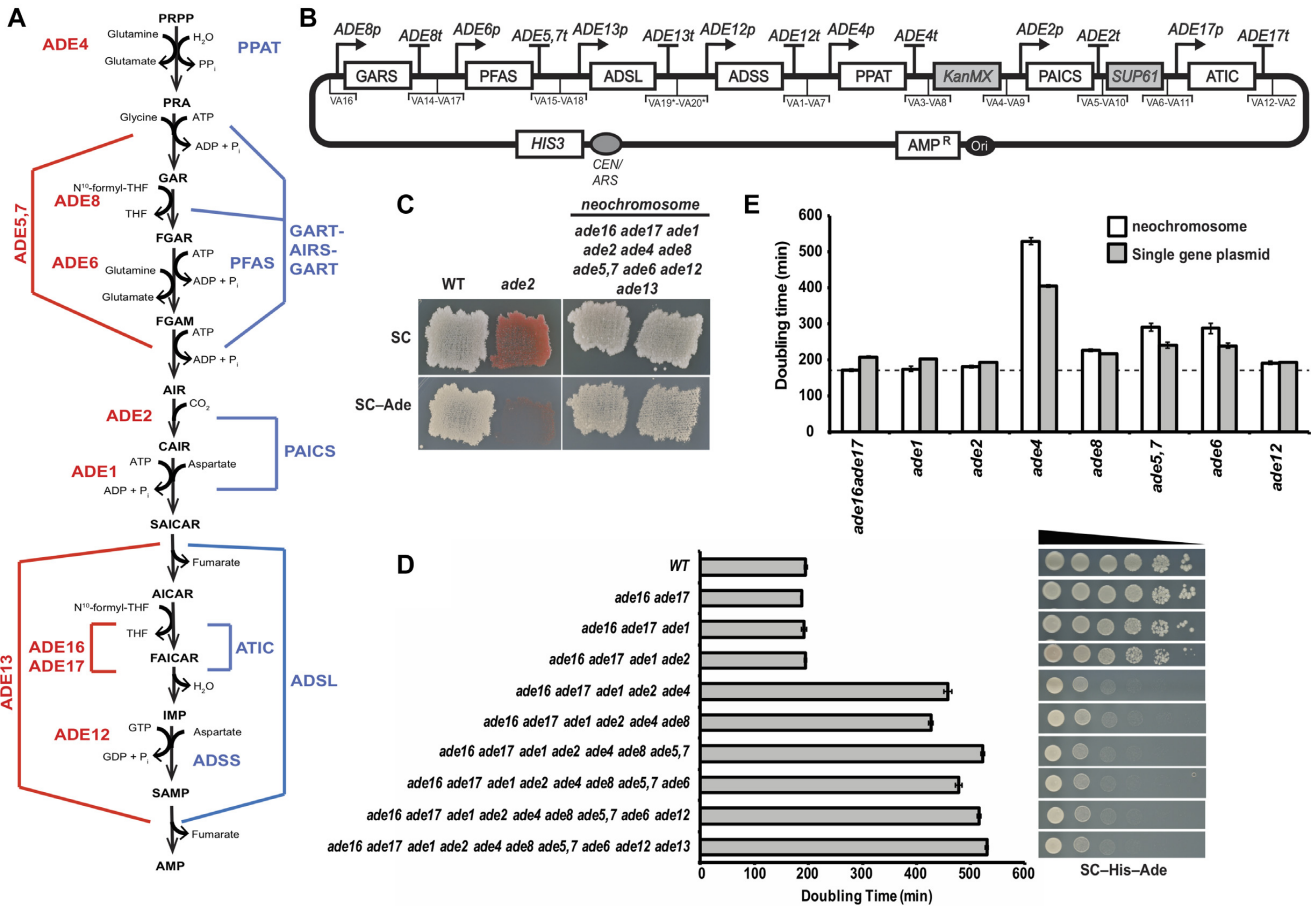
Yeast and human adenine *de novo* pathway and humanized strain construction. (**A**) Schematic representation of the adenine de-novo biosynthesis pathway. Red, yeast genes; blue human. (**B**) Complete design of the human Purine de-novo Neochromosome for expression in yeast cells. Using yeast golden-gate assembly (18,22) we cloned each human gene (synthesized codon optimized for expression in *S. cerevisiae*) with its yeast ortholog's promoter and terminator and appropriate adaptors for VEGAS assembly (VA1-VA20) (22) of the neochromosome (asterisk indicate previously unpublished VEGAS adaptors, for details see methods section). (**C**) Comparing growth on media with and without adenine shows complementation in the humanized strain deleted for all yeast genes and expressing all human genes from the neochromosome. Comparison is to a *ade2Δ* strain that cannot grow on media without adenine and a WT (wild type; BY4741) that can grow on both. (**D**) Graphic representation of doubling time in medium without adenine of strains with increasing number of native adenine *de novo* genes deleted carrying the humanized neochromosome. Grow assay indicates a dramatic increase in doubling time following *ADE4* deletion. Spot assay on the left shows a similar trend to the growth assay, showing a dramatic growth defect following deletion of *ADE4*. (**E**) Graphic representation of doubling time to verify incomplete complementation of *ade4* by its human ortholog *PPAT* by testing single gene deletions and comparing neochromosome complementation and single gene plasmids in medium without adenine. Dotted line represents doubling time of a wild-type strain grown in the same medium.

Plasmids used in this work are listed in Supplementary Table S5. All human genes were designed to be synthesized codon optimized for expression in *Saccharomyces cerevisiae* using BioPartsBuilder (http://biopartsbuilder.org). Human adenine *de novo* protein sequences were retrieved from NCBI: PPAT (NP_002694.3), PAICS (NP_006443.1), ATIC (NP_004035), GARS-AIRS-GART (NP_000810.1), PFAS (NP_036525.1), ADSL (NP_000017.1) and ADSS (NP_001117.2). CDS were designed with appropriate overhangs and restriction enzyme to allow for cloning using yGG assembly (18). Fragments were synthesized and cloned through Gen9Bio or GenScript. For expression in yeast, human genes were cloned into yGG acceptor vectors (18) flanked by their yeast orthologs promoter and terminator (Figure 1B).

### Deletion of all 10 adenine *de novo* pathway genes in yeast

To delete all 10 genes, we constructed two deletion plasmids for each gene using yGG assembly, using the same flanking regions used as regulatory elements to express the human genes (Supplementary Figure S1). Oligonucleotides used to amplify regulatory region from the yeast genome are listed in Supplementary Table S6. One plasmid insert consisted of a *URA3* gene flanked by each target gene's upstream and downstream flanking regions (500 bp upstream and 200 bp downstream), and was used to delete the target ORF via single step gene replacement. The second plasmid insert consisted of the same flanking sequences separated by a linker sequence (ATGGAGCATCTTTGCAAGGATCTTGCCACTGGAATGCGTAA), this insert fragment was used subsequently to delete each *URA3* gene using 5-FOA counter-selection (19). Due to the fact that ADE16 and ADE17 are redundant in their adenine auxotrophy, we started the process by deleting them in parallel in the two different mating types. The single mutants were crossed to construct a double heterozygous diploid that was then sporulated to form the double mutants. For the following deletions we deleted the genes consecutively: ade1, ade2, ade4, ade8, ade5,7, ade6 and ade12. For the only essential gene in the pathway, ade13 deletion, deleting the genes upstream in the pathway renders it nonessential, thus it can be deleted in the ade1 ade2 ade4 ade8 ade5,7 ade6 ade12 multi-deletion haploid strains. This methodology allowed sequential deletion of multiple target genes without employing multiple markers. This exercise was performed in both *MAT****a*** and *MATα* haploid strains. All Oligonucleotides used to verify deletion of the 10 genes are listed in Supplementary Table S6.

### Engineering of PPATs from different organisms

PPATs from different organisms were designed as described above and synthesized by Gen9Bio using yGG or using SGI-DNA BioXP 3200 system cloned in yeast. Briefly, PPAT CDS’s were synthesized (SGI-DNA) flanked by 70 bp of homology to and pAV115 pre-cloned with *ADE4* promoter and terminator flanking an RFP cassette flanked by *Bsm*BI sites (pNA0647). Plasmid was digested with *Bsm*BI and treated with CIP alkaline phosphatase (New England Biolabs, M0290L) recovered from gel using ZymonClean gel recovery kit (Zymo Research, D4002). It was co-transformed into *ade4Δ* cells with synthesized fragment. Transformants were screened for complementation on SC–Leu–Ade medium. For non-complementing constructs positive clones were screened on SC–Leu. For sequence verification plasmids were recovered from yeast as described below and transformed into bacteria.

### CRISPR–Cas9 system

CRISPR–Cas9 system was used to make point mutations and tag proteins. Cas9 expression plasmid was constructed by amplifying the Cas9 gene with *TEF1* promoter and *CYC1* terminator from p414-TEF1p-Cas9-CYC1t (20) cloned into pAV115(18) using Gibson assembly (21). gRNAs acceptor vector (pNA0304) engineered from p426-SNR52p-gRNA.CAN1.Y-SUP4t (20) to substitute the existing CAN1 gRNA with a *Not*I restriction site. gRNAs were cloned into the *Not*I site using Gibson assembly (21). For engineering yeast using the Cas9 system, cells were first transformed with the Cas9 expressing plasmid. Following a co-transformation of the gRNA carrying plasmid and a donor fragment. Clones are then verified using colony PCR with appropriate primers.

### Neochromosome engineering

Prior to assembly of a full neochromosome we engineered a transcription unit (TU) for each of the human genes flanked by their yeast orthologs regulatory elements and left and right VEGAS adaptors using yeast golden gate assembly (18,22) (Figure 1B). Operationally, we defined the orthologs’ regulatory regions as 500bp upstream and 200 bp downstream or up to the next gene boundary, whichever is shorter. The TUs were cloned into an acceptor vector (pAV10) that carries only a bacterial selection marker (Amp^R^) and *Not*I sites flanking the cloned TU.

Neochromosome assembly was performed in two steps. First, the *PPAT*, *PAICS* and *ATIC* TUs were assembled into a *LEU2* VEGAS vector (22) including a *kanMX* cassette and a *SUP61* cassette. The *kanMX* cassette was used to evaluate correct assembly by replica plating to YEPD supplemented with G418. The *SUP61* cassette was included to render the neochromosome essential in yeast strains lacking the single copy and essential gene tRNA-tS(CGA)C. Following transformation onto SC–Leu plates, replica plating to YEPD with G418 showed that 100% of the colonies were G418^R^ (compared to control transformations lacking either *KanMX* or *PAICS* that showed 10 times less colonies overall and either no or very few G418^R^ colonies, respectively). Ten colonies were verified by junction spanning PCR, and all were correct. One colony was picked to purify the neochromosome that was transformed into bacteria, and sequence verified by PacBio Sequencing.

The second step was cloning *GARS, PFAS, ADSL and ADSS* by replacing the *LEU2* marker with a *HIS3* marker (pNA0177). Briefly, in addition to the parts containing the transcription units, a part containing the *HIS3* marker with homology to the sequence flanking the *LEU2* marker was transformed, providing selection for assembly into the existing plasmid. Following transformation onto SC–His plates, replica-plating to KanMX and SC–Leu plates was performed. Of 30 transformants, two were G418^R^ and did not grow on SC–Leu, indicating successful ‘eSWAP-In’. Following verification of assembly using both junction spanning PCR tests, as well as additional PCR tests internal to TUs, the correct plasmids were purified from the yeast, transformed into bacterial cells and sequence verified.

### Plasmid recovery from yeast

Plasmid recovery from yeast was carried out using Qiagen buffers and Zyppy plasmid Miniprep kit (Zymo Research, D4037). Briefly, pelleted yeast cells from 1 mL of saturated culture were vortexed for 10 m in the presence of 100 μl of 0.5 mm glass beads and 250 μl of Qiagen P1 buffer (Qiagen, 19051) supplemented with 100 μg/ml RNAase A (Qiagen, 19101). Followed by addition of 250 μl P2 buffer (Qiagen, 19052), mix and addition of 350 μl buffer N3 (Qiagen, 19052). Following centrifugation for 10 m, supernatant was loaded on a Zyppy plasmid Miniprep kit column and washes were performed according to the manufacturer's instruction. Plasmid DNA was eluted using sterile water heated to 55°C. 10 μl of plasmid was transformed into competent TOP10 bacteria.

### Measuring doubling time in liquid culture

Saturated cultures were diluted 1:20 in the appropriate medium on a 96-well flat bottom plate. The plate was incubated at 30°C, and the *A*_600_ for each well was measured every 10 min for 48 h on a BioTek Eon plate reader. The plate was shaking for 30 s before each measurement. The resulting data containing the *A*_600_ of each time point of each well was exported in an Excel spreadsheet, and the maximum slope of the growth curve for each sample was calculated using the growth rate algorithm in R written by Danielle Carpenter from Princeton University (https://scholar.princeton.edu/sites/default/files/botsteinlab/files/growth-rate-using-r.pdf). The generation time of each strain was then calculated using the formula log(2)/growth rate, in which the growth rate was obtained from the algorithm mentioned above. At least 4 biological replicates and two technical replicates were tested for indicated strains.

### Function testing by complementation

To test for the function of genes in the adenine *de-novo* pathway we performed complementation tests. The examined cells were plated on or grown in appropriate media with or without adenine and their growth was compared to cells carrying their yeast orthologs.

### Isolation of PPAT suppressors

*ade4::PPAT* strains of both mating type were sequentially grown and mass-transferred in restrictive conditions (SC–Ade). First, we isolated single colonies and picked 96 of each mating type to grow to saturation (48 h.) in SC medium. We then diluted the cultures by 10^−3^-fold to SC–Ade and grown for 96 h, re-diluted 10^−3^-fold into SC–Ade. This was repeated four more times till most cultures were saturated after 48 h. In addition, from each mating type we sampled 8 of the cultures in each cycle to follow their evolvement through the experiment. Following the five cycles we isolated one single colony from each culture for genomic DNA preparation.

### Genomic DNA preparation

For genomic DNA preparation we used NORGEN Fungi/Yeast genomic DNA Isolation Kit (BIOTEK CORP.; 27300) following the manufacturer's instructions.

### Genomic sequencing of suppressors

Paired-end whole-genome sequencing was performed using an Illumina 4000 system and TruSeq preparation kits. In total, 35 samples were sequenced with 3.7M – 39.5M paired reads generated per sample. The length of each read was either 101 base pairs or 151 bp. Quality control was performed using FastQC version 0.11.2 software (24). All of the reads in the FASTQ format were aligned to the *S. cerevisiae* reference genome constructed starting with the sequence for control strains (strain BY4741 BY4742 genome sequences) using Burrows–Wheeler Aligner (BWA) version 0.7.8 software (25) with -P -M -R parameter settings. Approximately 95–99% of the reads were aligned to the corresponding reference genome. GATK version 3.2 software was used to do the preprocessing (mark duplicates and local re-alignment around indels) and variant calls with––genotyping_mode DISCOVERY –stand_emit_conf 10 –stand_call_conf 30 parameter settings (26–28). The results were subjected to a set of post-processing filters requiring: for SNPs, (i) a minimum of 10-fold coverage per variant site, (ii) WT reads in <10% of the total reads per site and (iii) reads supporting the variant of the control sample in <5% of the total reads per site; for Indels, (i) a minimum of 10-fold indel coverage per indel site of the treatment sample, (ii) a maximum of 5-fold indel coverage of the control sample (iii) a minimum of 90% of treatment indel within the local region using 50 bp flanking regions on both directions. snpEff version 4 software (29) was used to annotate each variant using Toronto-2012 gene GFF file.

### RNA preparation and sequencing

Cells were grown for 24 h in SC medium to saturation, then diluted into SC–Ade medium to a concentration of 10^7^ cells/ml. Samples were collected at 0, 1, 3 and 6 h, centrifuged and the cell pellets were flash frozen in liquid N_2_ to be stored at –80°C until RNA extraction.

RNA was prepared as described as in (30) from 10^7^ yeast cells by using the RNeasy Minikit (Qiagen; 74106) as per the manufacturer's instructions. In brief, cells were lysed enzymatically, and eluted RNA was treated with DNase (Qiagen; 79254) in solution before passage over a second column and elution in water. Approximately 5 ng of each sample was used for RNA amplification and library preparation using the CEL-Seq2 protocol taking only one-fifth of the amplified RNA to prepare the library. Paired end sequencing was performed on the Illumina NextSeq 500. The sequencing data was de-multiplexed using the CEL-Seq pipeline (31). Mapping of the reads was done using bowtie2 version 2.2.6 (32) as follows way: for WT samples the reads were mapped using the genome of BY4741, for the mutant samples the reads were mapped using the BY4741 genome adding the human gene, PPAT. Read counting was performed using an adaptation of HTseq to count each UMI only once (31). The counts were normalized by dividing the total number of unique transcripts for each sample and multiplying by one million [Transcript per million (TPM)].

### Metabolomic analysis

Cells were switched from SC+ 20 mg/l adenine to SC–Ade for 1, 3, 6 h and then extracted with 75% ethanol. Supernatant (1 ml) was collected, vacuum dried, and stored at –80°C. The metabolite analysis was performed by LC–MS/MS on a Shimadzu Prominence LC20/SIL-20AC HPLC coupled to a ABSCIEX 3200 QTRAP triple quadrupole mass spectrometer as described previously (33). Chromatographic separation was performed using a C18-based column with polar embedded groups (Synergi Fusion, 150 × 2.0 mm 4 μ, Phenomenex). Infusion quantitative optimization was performed to acquire optimal product ion mass for each metabolite. Multiple reaction monitoring (MRM) was used to detect and quantitate metabolites. The two most abundant daughter ions were used when possible and metabolite peak area was normalized to total ion content. Buffers for positive-mode analysis were formic acid method (buffer A: 99.9% H_2_O/0.1% formic acid and buffer B: 99.9% methanol/0.1% formic acid), and ammonium acetate method (buffer A: 5 mM ammonium acetate in H_2_O and buffer B: 5 mM ammonium acetate in 100% methanol). TBA method (buffer A: 5 mM tributylamine (TBA) and buffer B: 100% methanol) was used for negative-mode. The area under each peak was quantitated by Analyst software, and normalized against total ion count (Supplementary Table S3).

### MG132 treatment

In order to increase membrane permeability to MG132 we deleted *erg6* in the Ade4 versus Ppat expressing cells (34). Calbiochem MG132 was purchased from Sigma-Aldrich (Millipore-Sigma, 474790). 10 μM MG132 was used in plates and 50 μM MG132 was used in liquid medium.

### Immunoblot analysis

Because there are antibodies available for neither Ade4 nor Ppat we used V5 tagged protein for all immunoblot experiments presented in this work. To optimize the tagging strategy for each protein we tagged both ends and chose those tags that did not decrease the activity of the protein as measured by growth on medium without adenine in an *ade4* deletion background (Supplementary Figure S2). Surprisingly, Ade4p could only be tagged in an active form on its C terminal whereas PPAT protein was only active when tagged on its N terminal. Chimeric proteins were tagged on their N-ter similarly to Ppat. For protein extraction cells expressing either Ade4-V5 or V5-Ppat were grown for 24 h. in SC medium, followed by dilution and transfer to media with or without adenine and with or without 1 μM estradiol as described above. 20 OD_600_ were collected, washed once with water and flash frozen in liquid nitrogen for storage in –80°C until protein preparation. Cells were incubated for 30 min. in NaOH buffer [150 mM NaOH, 2 mM DTT and 1× cOmplete™, EDTA free protease inhibitor Cocktail (Roche, 11873580001)] on ice for 30 min. Following 10 min centrifugation at 4°C, cells were lysed in 150 ml of lysis buffer [20 mM HEPES, pH 7.4, 0.1% Tween 20, 2 mM MgCl_2_, 300 mM NaCl, 1.5 mM DTT and 1× cOmplete, EDTA free protease inhibitor Cocktail] in the presence of equal volume of 0.5 mm glass beads (1:1 cell slurry:beads) by vortexing (10 min. at 4°C). Following centrifugation (10 200 rpm, 4°C, 3 min), 150 μl of clarified whole-cell extract was collected and total protein was measured using Bradford protein assay kit (BioRad, 5000006). For the estradiol experiment (Figure 3A), total cell lysate was concentrated using microcon-30 kDa filter unit (EMD Millipore, MRCF0R030) before measuring total lysate concentration. Samples were then mixed with 4× LDS sample buffer (Life Technologies, NP0007). Samples were heated at 70°C for 10 min and loaded onto either a 12% pre-cast Bis–Tris gel (Life Technologies, NP0342BOX) or a 10% pre-cast Bis–Tris gel (Life Technologies, WB1202BX10) and electrophoretically separated in 1× MOPS buffer (Life Technologies, B000102). Protein transfer was carried out using a BioRad Trans-blot Turbo Transfer system and corresponding reagents. Anti-V5 antibody was from Sigma Invitrogen (46-1157; mouse) and anti-*α*-Tubulin ((35), Rabbit) served as a control for loading. Secondary antibodies were from LI-COR (IRDye 800CW, 926-32210; IRDye 680RD, 926-68071). Western blots were developed and quantified using the LI-COR Odyssey and Image Studio Software.

### *ade13* suppressor mutation recessivity test

In order to distinguish whether these *ade13* suppressor mutants are hypomorphs or gain of function mutations we crossed each of the re-constructed strains to a *MATalpha* strain containing *ade4::PPAT* and a WT copy of *ADE13*. We then plated the cells on media with or without adenine. Comparing the growth of the *ade4::PPAT ade13* heterozygotes with *ade4::PPAT ADE13* WT homozygous on medium without adenine revealed that the mutants are recessive and thus most likely hypomorphs.

### DNA shuffling

We performed DNA shuffling as described by Stemmer (36) and modified (37). Briefly, single gene expression plasmids were used as templates to amplify the coding sequences of Human, Camel and Whale PPATs with *ADE4* promoter and terminator using forward primer AACGCTCGTAAGTAAATATTGATTTATAC (NA145) and reverse primer CAATTCTTTTATCTTCTTTTCTTTTTGTAC (NA146). PCR products were mixed in two separated reactions: 1) human with camel and 2) camel with Whale. In each reaction PCR products were mixed at a 1:1 ratio to a final concentration of 50 ng/μl in a final volume of 70 μl. DNA solution was cooled down to 15°C in a thermocycler and mixed with 30 μl of DNase I solution [100 ml DNase I 1:200 dilution (Qiagen, 79254)]. Following 5 min incubation, solution was transformed to a new tube containing 6 μl 0.5 M EDTA on ice. DNA was run on a 2% agarose gel and fragments 400 bp to 1 kb were cut from the gel and isolated using Zymoclean™ Gel DNA Recovery Kit (Zymo research, D4002). DNA was eluted from the column with 20 μl and water and run on a gel for verification. 10 μl purified fragmented DNA was subjected to assembly PCR (10 μl of 5× Phusion buffer, 2 μl of dNTPs soliton and 1 μl of Phusion polymerase) in a 50 μl reaction with the following cycling protocol: 96°C for 90 s; 35 cycles of (94°C, 30 s; 65°C, 90 s; 62°C, 90 s; 59°C, 90 s; 56°C, 90 s; 53°C, 90 s; 50°C, 90 s; 47°C, 90 s; 44°C, 90 s; 41°C, 90 s; 72°C, 4 m); 72°C, 7 m; 16°C thereafter. Following gel verification, showing a smear of assembled molecules running up to the original fragment size, Primer-containing PCR was done using 1 μl of the above DNA as template [20 μl 5× Phusion buffer, 2 μl dNTPs solution, 40 pmol each primer (NA145 and NA146), 3 μl DMSO and 5U Phusion polymerase] in a 100 μl reaction with 25 cycles and 2 min elongation time. Products of the DNA shuffling protocol were co-transformed with *Bsm*BI digested pNA0647 plasmid (pAV115(18) pre-cloned with *ADE4* promoter and terminator flanking an RFP cassette flanked by *Bsm*BI sites) into *ade4Δ* cells and selection was applied for fast growing variants on medium without adenine.

## RESULTS

### One-shot transplantation of 7 human genes for adenine synthesis into yeast

We wanted to investigate whether we can synthesize and transplant a functional multigene human pathway into yeast to replace the existing yeast pathway. Our working hypothesis was that, unlike single-gene substitutions, transplanting a multigene pathway might teach us about the regulation of the pathway and the interaction between the enzymes.

Full transplantation of a functional human pathway into *S. cerevisiae* likely requires expression of multiple genes, each individually transcribed in the appropriate context and expression level. To accomplish these goals, we expressed the human structural genes for *de novo* adenine biosynthesis, recoded for optimal expression in yeast and controlled by promoter and terminator regions from the cognate yeast genes. The human adenine *de-novo* pathway involves 12 steps, catalyzed by seven distinct proteins, some of which harbor more than one enzymatic activity (Figure 1A). We used a combination of yeast golden gate (yGG) assembly (18) to build individual transcription units (TUs) followed by versatile genetic assembly system (VEGAS) (22) to construct a full length neochromosome in two consecutive steps (see methods). Human CDSs were synthesized yGG compatible (18), and transcription units (TUs) were constructed with VEGAS adaptors (VA) as described (22) (Figure 1b). The correct final structure of the neochromosome (Figure 1B) was verified by DNA sequencing.

In parallel to assembling the humanized adenine *de-novo* pathway, we deleted the yeast genes involved in the pathway to prove that the full heterologous pathway was functional. The 12-step yeast adenine *de-novo* pathway is catalyzed by 10 yeast proteins (Figure 1A). We deleted all 10 genes from the yeast genome in a stepwise manner, first using *URA3* to precisely delete each ORF followed by *URA3* deletion using 5FOA selection (see Materials and Methods).

The neochromosome was transformed into the multi-deletion strain as well as each individual adenine auxotrophic mutant available, and growth in the absence of adenine was assessed (Figure 1C and Supplementary Figure S1). In the presence of adenine, no growth defects were observed. The humanized strain grew in the absence of adenine, demonstrating the transfer of the ability to synthesize purines *de-novo* in the complete absence of adenine supplemented from the medium. However, growth on this medium was substantially slower than the wild type. Our subsequent investigations centered on characterizing this growth defect and improving cross-species complementation by a transplanted metabolic pathway.

### A single gene, *PPAT*, accounts for only partial complementation of the human adenine *de novo* pathway in yeast

Compared to the wild-type (WT) strain, the fully humanized strain showed a significantly longer doubling time in adenine-free media. Since the yeast genes were deleted sequentially, we mapped the defect(s) by transforming the neochromosome into the series of multi-deletion strains. Examination of doubling times revealed that the most significant effect on growth resulted from deletion of *ade4* (Figure 1D). Because all strains grew equally well in the presence of adenine, and the slow growth phenotype was recessive, it suggested that slow growth reflected partial loss of function (a hypomorph), and not production of a toxic product. *ADE4* encodes phosphoribosylpyrophosphate amidotransferase. Like its mammalian and bacterial counterparts *PPAT* and *PurF*, *ADE4* uses PRPP (phosphoribosyl pyrophosphate) as a substrate (Figure 1A).

To verify that complementation by *PPAT* was responsible for the growth defect, the function of each human gene was tested in individual deletion mutants (Figure 1e). In agreement with the results of the multi-deletion strains, *PPAT* showed only partial function in an *ade4* deletion background. We attempted to increase the expression of *PPAT* by both copy number and promoter strength using a strong constitutive promoter (*ADH1* promoter). In all cases, there was no effect on growth in medium without adenine in the absence of *ADE4* and the presence of *PPAT* (Supplementary Figure S1). These results suggest that the observed partial complementation phenotype is not simply due to insufficient *PPAT* mRNA. Thus, we have shown that by transplanting a multigene pathway from human into yeast, we can expose the node in the pathway most sensitive to genetic perturbation. In the case of the *de novo* pathway of adenine synthesis in human, it is the first step of the pathway, catalyzed by Ppat that seems to be most sensitive.

### Suppressor analysis reveal host genes can improve cross species transplantation complementation

**Figure 2.**
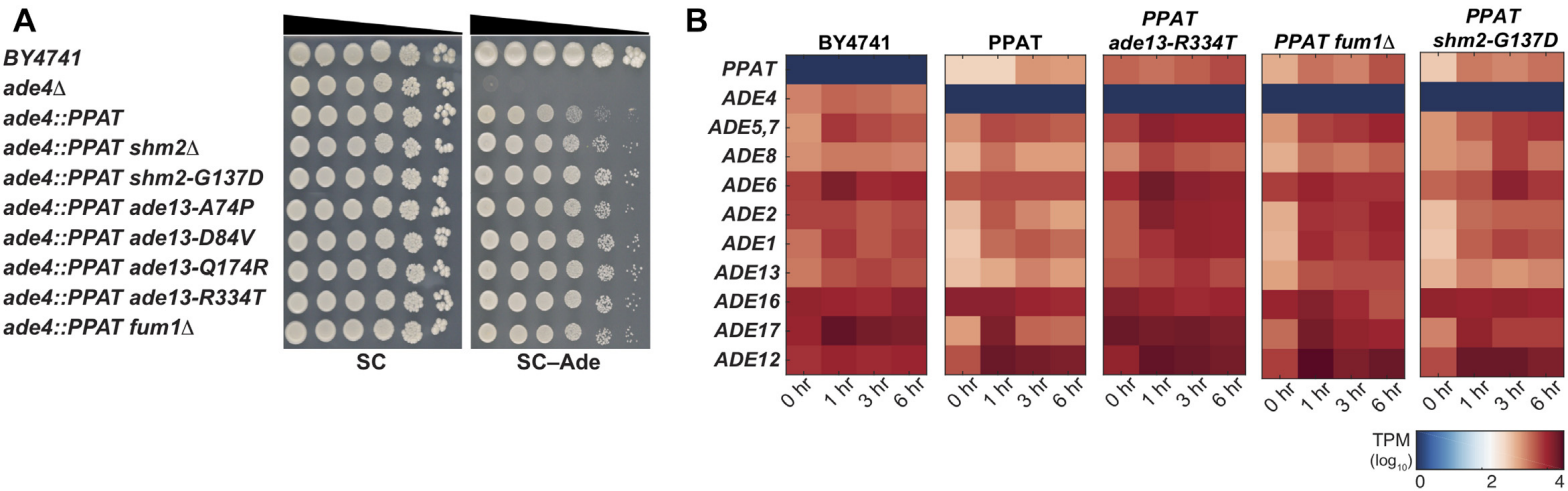
*PPAT* suppressor analysis. (**A**) Reconstitution of mutations found in suppressor strain genomes. All reconstructed suppressors alleviate the PPAT phenotype. Comparing mutants to deletion strain indicates that in all suppressors the phenotype is probably due to loss of function mutations. (**B**) Heat-map showing *ADE* gene transcripts in the yeast strains tested (BY4741, *ade4::PPAT*, *ade4::PPAT ade13-R334T*, *ade4::PPAT fum1Δ* and *ade4::PPAT shm2-G137D*) grown in SC and moved to SC–Ade. Sampled were collected after 1, 3 and 6 h for RNA preparation. RNA-seq analysis was performed on all samples and results are presented in a heat-map (scale represents log_10_, transcripts per million [tpm]).

Following the identification of *ADE4/PPAT* as the node most sensitive for cross-species transplantation, we wanted to examine whether we could identify any yeast factors that might improve complementation. We hypothesized that, given enough time under restrictive conditions, yeast cells would accumulate mutations beneficial for the growth in the tested condition. In this case, cells that are deleted for *ade4* and express *PPAT* (*PPAT* strain) grow slowly in medium without adenine. Thus, any mutation that can improve the growth in these conditions will take over the cell population, allowing identification of mutations that can improve growth in adenine-free medium. We isolated a large number of independent spontaneous suppressors of the *PPAT* partial complementation phenotype by mass-transferring and growing hundreds of independent populations of cells in adenine-free medium. We then picked the strongest suppressors (Supplementary Figure S3) and performed whole-genome sequencing (WGS) on 36 of them. The mutations, none of which mapped to *PPAT*, identified in each suppressor are listed in Supplementary Table S1. Previous work showed that adenine starvation is highly mutagenic in yeast (38), potentially accounting for the high number of mutations found in some of these suppressor strains. Recurrent mutations were subsequently introduced individually into a clean *PPAT* strain to identify those sufficient to confer enhanced growth on adenine-free medium. In agreement with the literature (23,39–41), multiple recurrent mutations were mapped to genes with roles affecting (S)AICAR levels. (S)AICAR are intermediates in the adenine *de-novo* pathway that were shown in yeast to bind Bas1 and Pho2, transcription factors that induce the expression of all *ADE* genes (23). Recessive loss of function mutations in *FUM1* (fumarase) and *SHM2* (Cytosolic serine hydroxymethyltransferase), and partial loss of function mutations (Figure 2A and Supplementary Figure S4) in *ADE13* (Adenylosuccinate lyase), are all consistent with elevated levels of (S)AICAR (Figure 2A and Supplementary Figure S4). In addition, we performed transcriptome analysis on *PPAT* cells carrying some of the mutants found in the screen (Figure 2B). Similar to the results obtained by Rebora *et al.* (40) for *ade13* mutants, all three mutants tested have higher basal levels of mRNAs for most *ADE* genes as well as *PPAT*, and also much higher induction of *ADE* genes in response to adenine deprivation. These results, together with our findings related to *PPAT* overexpression, indicate that overexpression of *PPAT* alone is insufficient to fully complement *ADE4*. However, our analysis suggests that this can be partially alleviated through a feed forward loop regulated by pathway intermediates. We believe that in yeast cells Ppat acts as a bottleneck that supplies small amounts of its product (PRA) to the downstream steps. But, in the presence of suppressors that increase the positive feedback on the downstream steps, there is an increase of PRA utilization and thus modestly increased production of the pathway end products. These results show that in cases where there is a measurable phenotype, suppressor analysis combined with multiple ‘omics approaches can improve cross-species complementation and provide important insights into pathway regulation.

### Phylogenetic analysis of PPATs reveals correlation between phylogenetic distance and complementation in yeast cells

Besides transcription regulation, a common feature of most metabolic pathway regulation is allosteric inhibition at key regulatory nodes by pathway metabolites (12). Studies in human, yeast and bacteria have shown that there is allosteric inhibition of the *ADE4/PurF/PPAT* active site through pathway end products (41–43). In addition, *in vitro* tests of the activity of PurF from *E. coli* and its *B. subtilis* ortholog revealed inhibition by distinct nucleotides (42). We thus hypothesize that inhibition of the human protein, Ppat, is higher than Ade4 in yeast cells, leading to only partial complementation. Given that we did not identify any *PPAT* mutation in our suppressor screen, we postulated that preserving the activity of the protein while elevating the allosteric inhibition requires a complex set of variations in the protein sequence.

### Inhibition of human PPAT is higher than Ade4 in the conditions of a yeast cell

**Figure 3.**
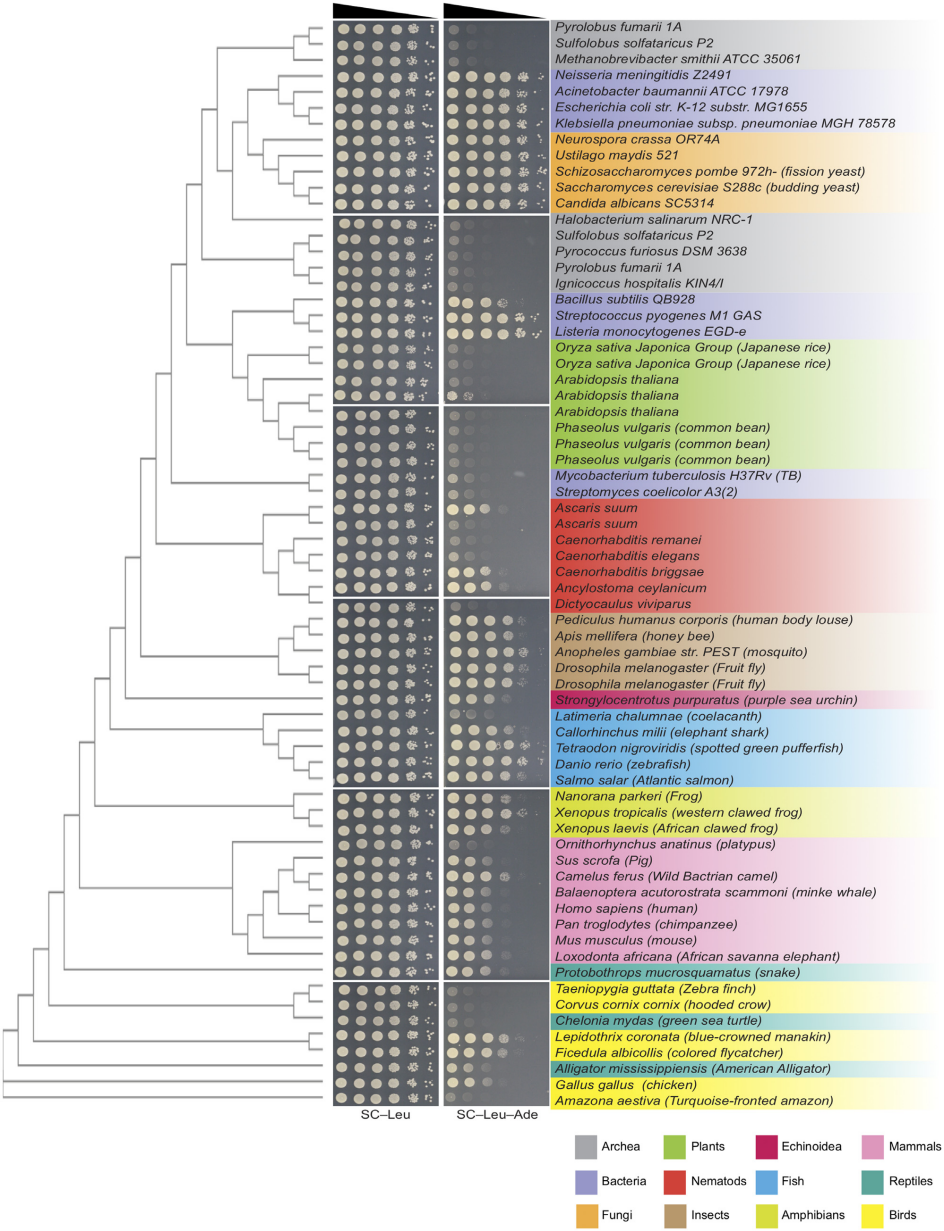
Phylogenetic analysis of *PPAT*s. Phylogenetic tree generate by multiple sequence alignment CLUSTAL-OMEGA (44) on the EMPL-EBI site (49,50) aligned with a spot assay showing the growth of *ade4Δ* strains expressing *PPAT*s from different organisms and their ability to complement *ade4* deletion.

Therefore, we decided to broaden our analysis and challenge cross-species transplantation to Ppats from the entire tree of life, assuming that we can identify trends in the phylogenetic tree that can help up improve the human *PPAT* function in yeast cells. We evaluated nearly 70 *PPAT* orthologs (Supplementary Table S2) by introducing the codon-optimized orthologs on a plasmid into *ade4*-deleted yeast cells and examining their growth properties on adenine-free media (Figure 3). A phylogenetic tree was generated based on a CLUSTAL-OMEGA multiple sequence alignment (44). Surprisingly, many organisms clustered in groups with respect to the extent of their complementation, and in general, these groups were correlated well in terms of phylogenetic relatedness. For example, Ppats from all plants and Archaea tested failed to complement, whereas all insect enzymes tested complemented almost completely. In bacteria, all gram-negative species tested complemented completely; whereas gram-positive species showed variable function levels. Most mammals complemented similarly to human *PPAT*, except for platypus, which failed to complement, and Bactrian camel (‘camel’), which complemented much better than the other mammals (see below). Among the other chordates tested, all amphibians and fish complemented whereas reptiles and birds showed variable complementation. Although there might be an underlying biological explanation for the differences among chordates, a more likely explanation for lack of complementation is incomplete or incorrect sequence assembly and/or annotation for the less thoroughly studied species. Those organisms in which the genome is well studied, and thus more accurately annotated, showed a very clear correlation between extent of complementation among the closely related species. From this analysis, we hypothesize that species that adapted to similar environments, i.e. have similar ‘lifestyles’, show similar levels of *PPAT* function which might indicate an adaptation of the enzyme to the cellular environment and the organism needs (see discussion).

### Phylogenetic differences can be harnessed to improve cross species transplantation

**Figure 4.**
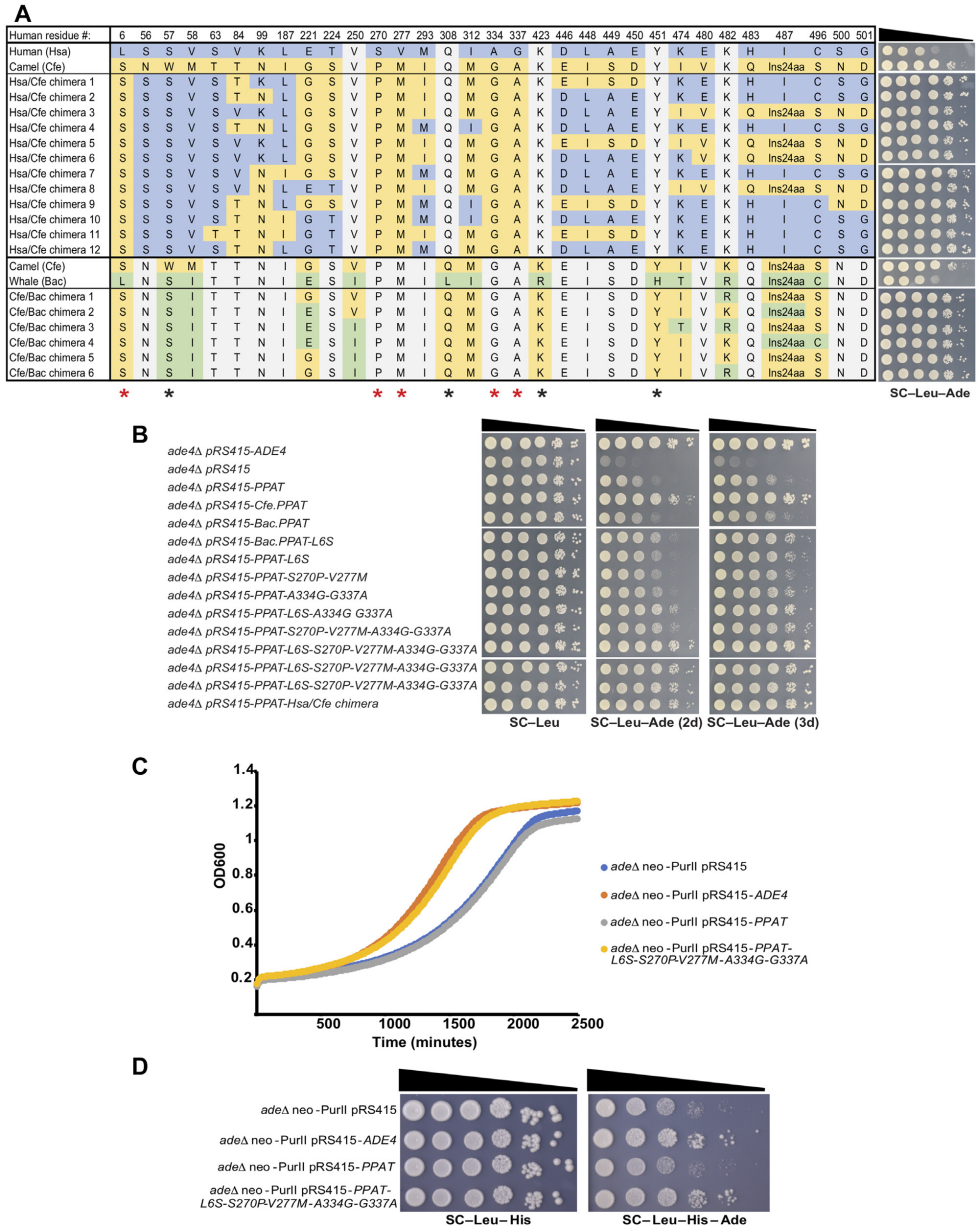
Phylogenetic analysis reveals distinct residues that affect PPATs function in yeast. (**A**) Left – Table showing the variable residues between human (Hsa), camel (Cfe) and whale (Bac) PPATs in the parental strain and in the DNA shuffling chimera products. DNA shuffling experiment was done in two separated reaction: Hsa + Cfe and Cfe + Bac. Colors represent the residues specific to each organism: Human (blue), camel (Yellow) and whale (green). Residues which are identical to both shuffled parents are marked in gray. Asterisks on the bottom indicate residues common to all chimeras, red asterisks are those absent from the human sequence. Right – Spot assay of *ade4Δ* strains carrying plasmids expressing the products of DNA shuffling between human (Hsa) and camel (Cfe) *PPAT*s and camel and whale (Bac) on SC–Leu/SC–Leu–Ade. All 18 candidates sequenced show 5 invariable common amino acids changes L6S, S270P, V227M, A334G and G337A (relative to the human reference sequence). (**B**) Growth assay of *ade4Δ* strains carrying plasmids expressing different version of *PPAT*s on SC–Leu/SC–Leu–Ade. We have reconstructed the five substitutions that were invariant in all DNA shuffling products into the human PPAT sequence in different combinations. Results shows that several of the combinations show increase complementation. However, the five substitutions variants (pRS415-PPAT-L6S-S270M-V227M-A334G-G337A) shows similar complementation as the Has/Cfe chimera. (**C**, **D**) Graphic representation of the growth in medium without adenine and spot assay of fully humanized yeast cells supplemented with either an empty vector (pRS415), vector expressing yeast *ADE4* gene (pRS415-*ADE4*), vector expressing human *PPAT* (pRS415-*PPAT*) or vector expressing human *PPAT* with the five substitution variant (pRS415-*PPAT*-L6S-S270M-V227M A334G G337A). Indicating that PPAT carrying the 5 substitutions complement as well as yeast *ADE4*.

As mentioned above, our motive for phylogenetic analysis was to identify key sequence variations that might improve human Ppat function in yeast. Camel *PPAT* functioned much better than the other mammalian *PPAT*s tested (Figure 3). There are 38 aa differences between the protein sequences of camel and human *PPAT*. Additionally, Minke whale (‘whale’) and camel *PPAT* differ at only 12 aa even though the whale, like human, functioned poorly. To identify the residues responsible for the differences we utilized DNA shuffling, which allowed the generation of chimeras between human / camel and whale/camel sequences (see Methods), with the pairs of template DNAs provided in equal proportions (36). We isolated 18 chimeric products that showed elevated complementation in an *ade4Δ* strain grown in adenine-free medium; 12 of them represented camel/ human chimeras and six were camel/whale chimeras (Figure 4A). All 18 chimeras complemented better than either parental gene. Sequencing of *PPAT* in those strains revealed that of the 55 variable residues among the three parental sequences, nine were shared among all chimeras: S6, S57, P270, M277, Q308, G334, A337, K423, Y451. Only five of these shared residues are absent from human *PPAT* and are represented by the following substitutions: L6S, S270P, V277M, A334G and G337A (Figure 4). The latter four are located in the PRTase domain of the enzyme. L6S is a substitution seen in three separate species of camels, suggesting this is not a sequencing error or a mutation that arose in cloning. L6S does not lie within the PRTase domain defined by the *E. coli* protein structure (PDB 1ECB), but forms part of an N-terminal extension common to all mammalian Ppats but absent from bacterial, fungal, and *Caenorhabditis elegans* Ppats (Supplementary Figure S5). This observation is consistent with the existence of a regulatory N-terminal extension in mammalian Ppats. This N-terminal tail is highly conserved among mammals, and its’ richness in glutamic acid residues suggests it might play a role in interaction with positively charged molecules (45).

To further verify these DNA shuffling findings, we reconstituted the 5 shared substitutions missing from human Ppat into it. We made all five substitutions as well as various combinations of them and one substituting into the whale Ppat (L6S). Analysis of their function shows that in most combinations there is some enhancement of function compared to native human Ppat (Figure 4B and Supplementary Figure S4). However, the strongest effect was seen with the 5-substitution variant, which showed function comparable to the chimera. Although not all combinations of substitutions were tested, due to their presence in all DNA shuffle chimeric products, we conclude that these five substitutions were necessary and sufficient to confer the phenotype. Both the 5-substitution human Ppat and the chimera complement substantially better than the camel Ppat (*cfe.PPAT*), which can probably be attributed to the single invariable residue missing for the camel sequence, S57. Together, these results support the contribution of nine specific residues to the activity of mammalian *PPAT*s as well as our ability to identify these important residues using the yeast system. The fact that both the chimera and 5-substitution human *PPAT* still grow slightly slower than cells expressing *ADE4* (Figure 4B and Supplementary Figure S4) suggests that additional residue changes might still enable full complementation.

Finally, we introduced *PPAT* with the five chimeric substitutions (L6S, S270P, V277M, A334G and G337A) into the fully humanized strain (*adeΔ* neo-purII). Figure 4C and D shows both growth curves as well as spot assays, indicating that *PPAT* carrying the five substitutions complements as well as *ADE4* in medium lacking adenine.

### Protein level correlates with the function of PPAT in yeast

**Figure 5.**
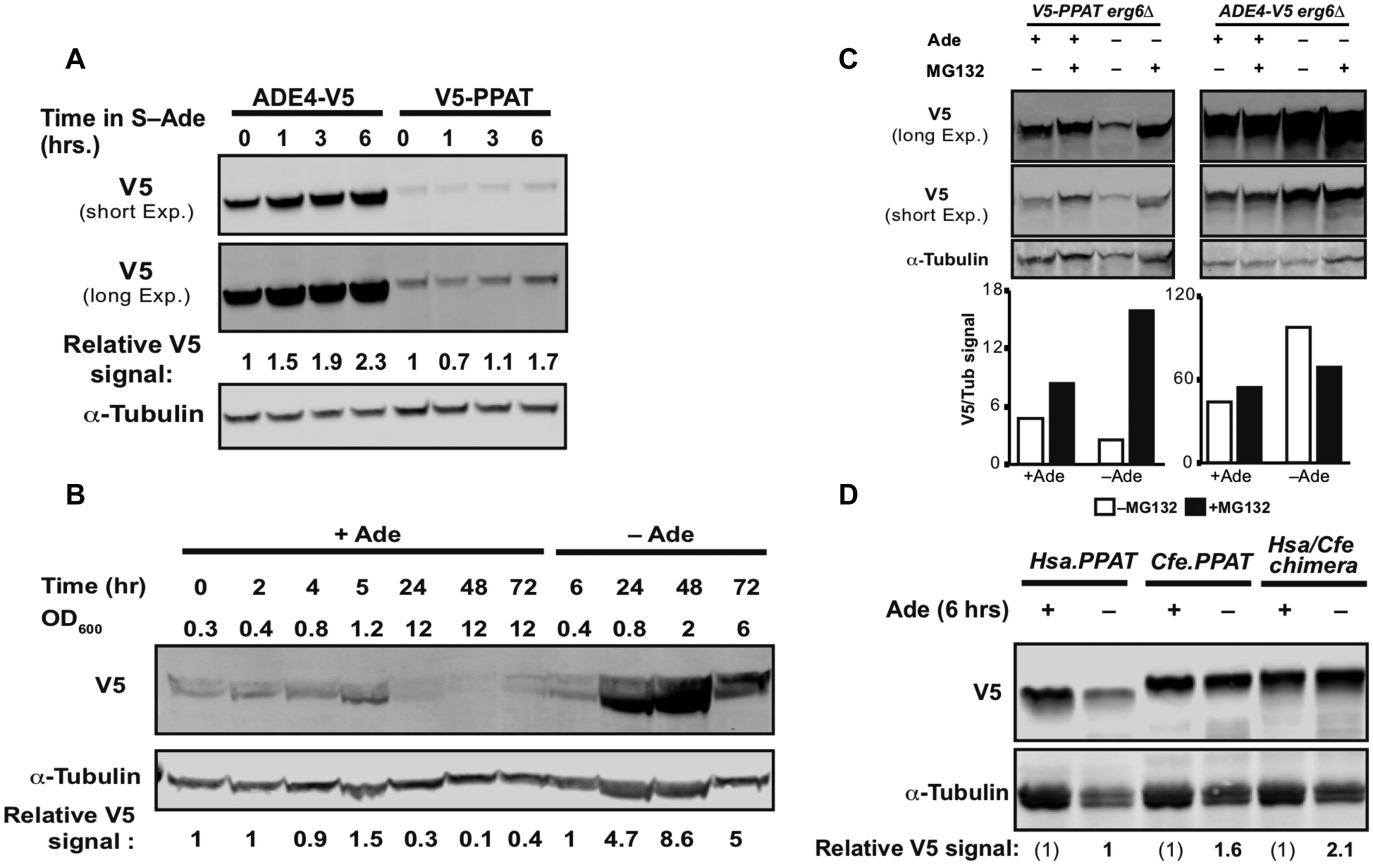
Ppat protein level is sensitive to presence of adenine in the medium. (**A**) Immunoblot of cells expressing either Ade4 tagged with V5 or Ppat tagged with V5 integrated into the native *ADE4* locus and transcribed from its native promoter. Both Ade4-V5 and V5-Ppat show an increase in protein level during prolonged time in media without adenine. Relative V5 signal is calculated V5/α-Tubulin divided by the signal in time 0 h. (**B**) Immunoblot of cells expressing Ppat tagged with V5 in medium with or without adenine. Relative V5 signal is calculated V5/α-Tubulin similar to (a). Accumulation of significantly more Ppat in media without adenine. Results were repeated in two biological replicates. (**C**) Immunoblot of cells expressing either Ppat or Ade4 tagged with V5 6 h in medium with or without adenine and with or without 50 μM MG132. Ade4-V5 expressing cells show similar levels of protein in the presence and absence of MG132, while V5-PPAT expressing cells show an increase in protein level in the presence of MG132 without adenine. The graph indicates the signal for V5 divided by the α-tubulin signal. (**D**) Immunoblot of cells expressing either Human Ppat (Hsa.PPAT), Camel PPAT (Cfe.PPAT) or Human/Camel chimera PPAT (Hsa/Cfe.PPAT) tagged with V5 expressed from a CEN/ARS plasmid regulated by *ADE4* native promoter and terminator. Cells were grown for 6 h in either SC–Leu or SC–Ade–Leu medium to maintain the expression plasmid. Samples were collected and total protein was prepared. Relative V5 signal is calculated as V5/α-Tubulin divided by the signal in Hsa.PPAT sample grown in SC–Ade media. Camel PPAT and chimera PPAT variants show higher levels of protein in adenine depleted medium.

Phylogenetic analysis has revealed key residues that have a strong effect on the function of human *PPAT* in yeast. However, it is still unclear how these residues affect the function of the protein. We thus decided to examine Ppat protein level in yeast cells. Given the lack of good antibody to detect Ppat, we tagged the protein (see methods). First, we examined the level of Ppat compared to Ade4 (tagged with the same tag) in adenine-free media. Surprisingly, throughout the experiment Ade4 levels were significantly higher than Ppat levels (Figure 5A), despite expression of both from the same genomic location flanked by identical regulatory sequences (see methods). Thus, we hypothesized that one possible explanation could be post translational modification leading to degradation of the human protein. We have discussed above the presence of allosteric inhibition on Ppat by pathway products. Previous reports have also shown that binding of nucleotides to Ppat causes a conformational change (42,43,46). Thus, a possible explanation for the reduction of Ppat protein level in yeast cells could be targeting of inactive protein for degradation. In the presence of adenine in the media, purine salvage pathway is responsible for converting adenine to AMP, IMP, and GMP. However, in media without adenine the *de novo* pathway converts PRPP into these products. If allosteric inhibition is involved in protein degradation, Ppat levels should increase only in the absence of the products, i.e. in adenine-free media. To decouple the positive feedback transcription regulation on *ADE* promoters from protein expression, we expressed *PPAT* under the control of an inducible promoter (see methods), so that RNA levels are affected by the inducer and not by media composition. Protein level analysis clearly shows (over 24 h), significantly more accumulation of Ppat protein in adenine-free relative to adenine-replete media (Figure 5B). This suggests a mechanism by which Ppat protein is sensitive to the normal intracellular nucleotide levels present in yeast cells. However, after 24 h of growth under adenine-depleted conditions, the inhibitory nucleotides levels are reduced sufficiently to alleviate inhibition and thus allow for protein accumulation. This work was supplemented by an examination of the metabolome, which showed a drop in most pathway products (AMP and IMP), except for GMP, as well as a drop in PRPP in the *ade4::PPAT* strain under adenine depletion conditions (Supplementary Figure S6 and Table S3). Thus, suggesting that GMP levels together with low PRPP levels might be sufficient to induce Ppat degradation. Collectively, this analysis is consistent with a mechanism that sensitizes human Ppat to yeast's steady-state level of inhibitory nucleotide(s) that binds to the protein, inactivates it, and induces degradation.

The most common form of protein degradation is via the 26S proteasome. To test whether allosterically inhibited Ppat is targeted for proteasomal degradation we examined the function of Ppat in the presence of proteasome inhibitor MG132. Ppat protein levels were higher in adenine-free medium supplemented with MG132 (Figure 5C). In addition, MG132-permeable (*erg6Δ*) *PPAT* cells grown in adenine-free media show faster growth compare to non-permeable cells on MG132 medium and permeable cells grown on control medium (without MG132) (Supplementary Figure S7). These results indicate that proteasomal degradation controls the Ppat level in yeast cells.

Finally, given the correlation between human Ppat protein level and its function in yeast cells, we examined whether the residues identified in our shuffle experiment affect protein level. We tagged both the camel and chimera Ppat's and examined protein levels in adenine-free medium. Both the camel and the Hsa/Cfe chimera strain show higher Ppat protein levels than human Ppat under adenine-depleted conditions (Figure 5D). This indicates that the increased growth of these Ppat variants on adenine-free media results from increased stability of the protein under adenine-depleted conditions.

Thus, biochemical analysis of human Ppat in yeast cells points to sensitivity of the human protein to the yeast steady-state intracellular nucleotide leading to post-translational regulation via proteasomal degradation, a regulation that can be controlled by changing specific residues near the active site (as defined in the *E. coli* protein structure).

## DISCUSSION

We report the first multigene transplantation of a human metabolic pathway into yeast. By a combination of synthesis and cloning methods, we engineered the functional expression of seven human genes of the complete adenine *de novo* biosynthesis pathway in yeast cells (Figure 1A). We show that it can partially support growth of yeast cells deleted for the corresponding orthologs on adenine-free media. This growth defect was linked to the reaction catalyzed by the human ortholog *PPAT*, representing the main regulatory node. We isolated suppressors that alleviate the phenotype by selecting for improved function of the entire pathway. Phylogenetic analysis enabled us to isolate specific residues in the enzyme's active site, which improved the function of the human protein by stabilizing the protein in yeast cells.

Our results suggest that the regulation of metabolic pathways adapts to the cellular environment. The adenine *de novo* pathway is regulated by both positive and negative feedback loops. The positive regulation is a feedforward transcription activation of genes in the pathway in response to accumulation of specific pathway intermediates and has been studied extensively in yeast cells (39–41,47). The negative feedback loop is induced by the product of the pathway that binds to the enzyme catalyzing the first committed step, Ppat, and allosterically inhibits substrate binding. This latter regulation was studied *in vitro* on the human (46), yeast (48) bacterial proteins (42). Cross-species transplantation exposed clear differences between the human and yeast phosphoribosylpyrophosphate amidotransferase proteins. We found that these differences are not restricted to human and yeast, and that orthologs from throughout the tree of life (Figure 3) show variable levels of complementation in yeast cells. Interestingly, species with similar ‘life styles’ show similar levels of function in yeast cells. As an organism settles into its niche it evolves to utilize available resources to accommodate its metabolic needs. This process provides selective pressure to increase fitness. In order to enable essential metabolic tasks in different environments, orthologs evolve to support the cell's need while maintaining their enzymatic activity. Thus, we suggest that the underlying differences in function between orthologs, as seen by cross species transplantation, might reflect the organism's metabolic state and the relative abundance and balance of key cellular metabolites to which this critical enzyme is tuned. This is the first *in vivo* experiment implicating that the level of key steady state metabolites differs between distantly related organisms, and that this level influences pathway regulation and protein adaptation.

We have used the adenine *de novo* pathway as a proof of concept for cross-species transplantation. We imagine that a similar strategy would apply for multigene pathway transplantation from any origin. We acknowledge that additional hurdles will probably need to be overcome as pathways are engineered in the future. However, we see this work as a springboard for future engineering of even larger cellular networks that can be dissected using phylogenetic analysis in yeast cells. The great geneticist Dobzhansky said ‘nothing in biology makes sense except in the light of evolution’. This work is a testament to the power of both evolution and the combination of modern and classical tools to understand fundamental processes have eluded us until now.

## Supplementary Material

gkz1098_Supplemental_Files

## References

[1] Zhang N., Osborn M., Gitsham P., Yen K., Miller J.R., Oliver S.G. Using yeast to place human genes in functional categories. Gene. 2003; 303:121–129. 12559573 10.1016/s0378-1119(02)01142-3

[2] Kruger W.D., Cox D.R. A yeast system for expression of human cystathionine beta-synthase: structural and functional conservation of the human and yeast genes. Proc. Natl. Acad. Sci. U.S.A. 1994; 91:6614–6618. 8022826 10.1073/pnas.91.14.6614 PMC44253

[3] Marini N.J., Gin J., Ziegle J., Keho K.H., Ginzinger D., Gilbert D.A., Rine J. The prevalence of folate-remedial MTHFR enzyme variants in humans. Proc. Natl. Acad. Sci. U.S.A. 2008; 105:8055–8060. 18523009 10.1073/pnas.0802813105 PMC2430358

[4] Lee M.G., Nurse P. Complementation used to clone a human homologue of the fission yeast cell cycle control gene cdc2. Nature. 1987; 327:31–35. 3553962 10.1038/327031a0

[5] Brachmann R.K., Vidal M., Boeke J.D. Dominant-negative p53 mutations selected in yeast hit cancer hot spots. Proc. Natl. Acad. Sci. U.S.A. 1996; 93:4091–4095. 8633021 10.1073/pnas.93.9.4091 PMC39492

[6] Kachroo A.H., Laurent J.M., Akhmetov A., Szilagyi-Jones M., McWhite C.D., Zhao A., Marcotte E.M. Systematic bacterialization of yeast genes identifies a near-universally swappable pathway. Elife. 2017; 6:e25093. 28661399 10.7554/eLife.25093 PMC5536947

[7] Hamza A., Tammpere E., Kofoed M., Keong C., Chiang J., Giaever G., Nislow C., Hieter P. Complementation of yeast genes with human genes as an experimental platform for functional testing of human genetic variants. Genetics. 2015; 201:1263–1274. 26354769 10.1534/genetics.115.181099 PMC4649650

[8] Kachroo A.H., Laurent J.M., Yellman C.M., Meyer A.G., Wilke C.O., Marcotte E.M. Evolution. Systematic humanization of yeast genes reveals conserved functions and genetic modularity. Science. 2015; 348:921–925. 25999509 10.1126/science.aaa0769 PMC4718922

[9] Truong D.M., Boeke J.D. Resetting the Yeast Epigenome with Human Nucleosomes. Cell. 2017; 171:1508–1519. 29198523 10.1016/j.cell.2017.10.043 PMC5732057

[10] Choi B.K., Bobrowicz P., Davidson R.C., Hamilton S.R., Kung D.H., Li H., Miele R.G., Nett J.H., Wildt S., Gerngross T.U. Use of combinatorial genetic libraries to humanize N-linked glycosylation in the yeast Pichia pastoris. Proc. Natl. Acad. Sci. U.S.A. 2003; 100:5022–5027. 12702754 10.1073/pnas.0931263100 PMC154291

[11] Kuijpers N.G., Solis-Escalante D., Luttik M.A., Bisschops M.M., Boonekamp F.J., van den Broek M., Pronk J.T., Daran J.M., Daran-Lapujade P. Pathway swapping: Toward modular engineering of essential cellular processes. Proc. Natl. Acad. Sci. U.S.A. 2016; 113:15060–15065. 27956602 10.1073/pnas.1606701113 PMC5206561

[12] Chubukov V., Gerosa L., Kochanowski K., Sauer U. Coordination of microbial metabolism. Nat. Rev. Microbiol. 2014; 12:327–340. 24658329 10.1038/nrmicro3238

[13] Gabaldon T., Koonin E.V. Functional and evolutionary implications of gene orthology. Nat. Rev. Genet. 2013; 14:360–366. 23552219 10.1038/nrg3456 PMC5877793

[14] Placzek S., Schomburg I., Chang A., Jeske L., Ulbrich M., Tillack J., Schomburg D. BRENDA in 2017: new perspectives and new tools in BRENDA. Nucleic Acids Res. 2017; 45:D380–D388. 27924025 10.1093/nar/gkw952 PMC5210646

[15] Brachmann C.B., Davies A., Cost G.J., Caputo E., Li J., Hieter P., Boeke J.D. Designer deletion strains derived from Saccharomyces cerevisiae S288C: a useful set of strains and plasmids for PCR-mediated gene disruption and other applications. Yeast. 1998; 14:115–132. 9483801 10.1002/(SICI)1097-0061(19980130)14:2<115::AID-YEA204>3.0.CO;2-2

[16] Kaiser C., Michaelis S., Mitchell A., Laboratory Cold Spring Harbor Methods in Yeast Genetics: A Cold Spring Harbor Laboratory Course Manual. 1994; 1994 edn NY Cold Spring Harbor Laboratory Press.

[17] Sherman F. Getting started with yeast. Methods Enzymol. 2002; 350:3–41. 12073320 10.1016/s0076-6879(02)50954-x

[18] Agmon N., Mitchell L.A., Cai Y., Ikushima S., Chuang J., Zheng A., Choi W.J., Martin J.A., Caravelli K., Stracquadanio G. et al. Yeast Golden Gate (yGG) for efficient assembly of S. cerevisiae transcription units. ACS Synth. Biol. 2015; 4:853–859. 25756291 10.1021/sb500372z

[19] Boeke J.D., Trueheart J., Natsoulis G., Fink G.R. 5-Fluoroorotic acid as a selective agent in yeast molecular genetics. Methods Enzymol. 1987; 154:164–175. 3323810 10.1016/0076-6879(87)54076-9

[20] DiCarlo J.E., Norville J.E., Mali P., Rios X., Aach J., Church G.M. Genome engineering in Saccharomyces cerevisiae using CRISPR-Cas systems. Nucleic Acids Res. 2013; 41:4336–4343. 23460208 10.1093/nar/gkt135 PMC3627607

[21] Gibson D.G., Young L., Chuang R.Y., Venter J.C., Hutchison C.A., Smith H.O. Enzymatic assembly of DNA molecules up to several hundred kilobases. Nat. Methods. 2009; 6:343–345. 19363495 10.1038/nmeth.1318

[22] Mitchell L.A., Chuang J., Agmon N., Khunsriraksakul C., Phillips N.A., Cai Y., Truong D.M., Veerakumar A., Wang Y., Mayorga M. et al. Versatile genetic assembly system (VEGAS) to assemble pathways for expression in S. cerevisiae. Nucleic Acids Res. 2015; 43:6620–6630. 25956652 10.1093/nar/gkv466 PMC4513848

[23] Hurlimann H.C., Laloo B., Simon-Kayser B., Saint-Marc C., Coulpier F., Lemoine S., Daignan-Fornier B., Pinson B. Physiological and toxic effects of purine intermediate 5-amino-4-imidazolecarboxamide ribonucleotide (AICAR) in yeast. J. Biol. Chem. 2011; 286:30994–31002. 21757731 10.1074/jbc.M111.262659 PMC3162458

[24] Andrews S FastQC a quality-control tool for high-throughput sequence data. http://www.bioinformatics.babraham.ac.uk/projects/fastqc.

[25] Li H., Durbin R. Fast and accurate short read alignment with Burrows-Wheeler transform. Bioinformatics. 2009; 25:1754–1760. 19451168 10.1093/bioinformatics/btp324 PMC2705234

[26] Van der Auwera G.A., Carneiro M.O., Hartl C., Poplin R., Del Angel G., Levy-Moonshine A., Jordan T., Shakir K., Roazen D., Thibault J. et al. From FastQ data to high confidence variant calls: the Genome Analysis Toolkit best practices pipeline. Curr. Protoc. Bioinformatics. 2013; 43:doi:10.1002/0471250953.bi1110s43. 10.1002/0471250953.bi1110s43 PMC4243306 25431634

[27] DePristo M.A., Banks E., Poplin R., Garimella K.V., Maguire J.R., Hartl C., Philippakis A.A., del Angel G., Rivas M.A., Hanna M. et al. A framework for variation discovery and genotyping using next-generation DNA sequencing data. Nat. Genet. 2011; 43:491–498. 21478889 10.1038/ng.806 PMC3083463

[28] McKenna A., Hanna M., Banks E., Sivachenko A., Cibulskis K., Kernytsky A., Garimella K., Altshuler D., Gabriel S., Daly M. et al. The Genome Analysis Toolkit: a MapReduce framework for analyzing next-generation DNA sequencing data. Genome Res. 2010; 20:1297–1303. 20644199 10.1101/gr.107524.110 PMC2928508

[29] Cingolani P., Platts A., Wang le L., Coon M., Nguyen T., Wang L., Land S.J., Lu X., Ruden D.M. A program for annotating and predicting the effects of single nucleotide polymorphisms, SnpEff: SNPs in the genome of Drosophila melanogaster strain w1118; iso-2; iso-3. Fly (Austin). 2012; 6:80–92. 22728672 10.4161/fly.19695 PMC3679285

[30] Mitchell L.A., Boeke J.D. Circular permutation of a synthetic eukaryotic chromosome with the telomerator. Proc. Natl. Acad. Sci. U.S.A. 2014; 111:17003–17010. 25378705 10.1073/pnas.1414399111 PMC4260612

[31] Hashimshony T., Senderovich N., Avital G., Klochendler A., de Leeuw Y., Anavy L., Gennert D., Li S., Livak K.J., Rozenblatt-Rosen O. et al. CEL-Seq2: sensitive highly-multiplexed single-cell RNA-Seq. Genome Biol. 2016; 17:77. 27121950 10.1186/s13059-016-0938-8 PMC4848782

[32] Langmead B., Trapnell C., Pop M., Salzberg S.L. Ultrafast and memory-efficient alignment of short DNA sequences to the human genome. Genome Biol. 2009; 10:R25. 19261174 10.1186/gb-2009-10-3-r25 PMC2690996

[33] Laxman S., Sutter B.M., Shi L., Tu B.P. Npr2 inhibits TORC1 to prevent inappropriate utilization of glutamine for biosynthesis of nitrogen-containing metabolites. Sci. Signal. 2014; 7:ra120. 25515537 10.1126/scisignal.2005948 PMC4427238

[34] Lee D.H., Goldberg A.L. Selective inhibitors of the proteasome-dependent and vacuolar pathways of protein degradation in Saccharomyces cerevisiae. J. Biol. Chem. 1996; 271:27280–27284. 8910302 10.1074/jbc.271.44.27280

[35] Schatz P.J., Georges G.E., Solomon F., Botstein D. Insertions of up to 17 amino acids into a region of alpha-tubulin do not disrupt function in vivo. Mol. Cell Biol. 1987; 7:3799–3805. 3316988 10.1128/mcb.7.10.3799-3805.1987 PMC368037

[36] Stemmer W.P. DNA shuffling by random fragmentation and reassembly: in vitro recombination for molecular evolution. Proc. Natl. Acad. Sci. U.S.A. 1994; 91:10747–10751. 7938023 10.1073/pnas.91.22.10747 PMC45099

[37] Arnold F.H., Georgiou G. Directed evolution Library Creation: Methods and Protocols. 2003; Totowa, N.J Humana Press.

[38] Achilli A., Matmati N., Casalone E., Morpurgo G., Lucaccioni A., Pavlov Y.I., Babudri N. The exceptionally high rate of spontaneous mutations in the polymerase delta proofreading exonuclease-deficient Saccharomyces cerevisiae strain starved for adenine. BMC Genet. 2004; 5:34. 15617571 10.1186/1471-2156-5-34 PMC544876

[39] Pinson B., Vaur S., Sagot I., Coulpier F., Lemoine S., Daignan-Fornier B. Metabolic intermediates selectively stimulate transcription factor interaction and modulate phosphate and purine pathways. Genes Dev. 2009; 23:1399–1407. 19528318 10.1101/gad.521809 PMC2701576

[40] Rebora K., Desmoucelles C., Borne F., Pinson B., Daignan-Fornier B. Yeast AMP pathway genes respond to adenine through regulated synthesis of a metabolic intermediate. Mol. Cell Biol. 2001; 21:7901–7912. 11689683 10.1128/MCB.21.23.7901-7912.2001 PMC99957

[41] Daignan-Fornier B., Pinson B. 5-Aminoimidazole-4-carboxamide-1-beta-D-ribofuranosyl 5′-Monophosphate (AICAR), a highly conserved purine intermediate with multiple effects. Metabolites. 2012; 2:292–302. 24957512 10.3390/metabo2020292 PMC3901205

[42] Smith J.L. Glutamine PRPP amidotransferase: snapshots of an enzyme in action. Curr. Opin. Struct. Biol. 1998; 8:686–694. 9914248 10.1016/s0959-440x(98)80087-0

[43] Holmes E.W., McDonald J.A., McCord J.M., Wyngaarden J.B., Kelley W.N. Human glutamine phosphoribosylpyrophosphate amidotransferase. Kinetic and regulatory properties. J. Biol. Chem. 1973; 248:144–150. 4348202

[44] Sievers F., Wilm A., Dineen D., Gibson T.J., Karplus K., Li W., Lopez R., McWilliam H., Remmert M., Soding J. et al. Fast, scalable generation of high-quality protein multiple sequence alignments using Clustal Omega. Mol. Syst. Biol. 2011; 7:539. 21988835 10.1038/msb.2011.75 PMC3261699

[45] Zalkin H., Smith J.L. Enzymes utilizing glutamine as an amide donor. Adv. Enzymol. Relat. Areas Mol. Biol. 1998; 72:87–144. 9559052 10.1002/9780470123188.ch4

[46] Holmes E.W., Wyngaarden J.B., Kelley W.N. Human glutamine phosphoribosylpyrophosphate amidotransferase. Two molecular forms interconvertible by purine ribonucleotides and phosphoribosylpyrophosphate. J. Biol. Chem. 1973; 248:6035–6040. 4726295

[47] Ljungdahl P.O., Daignan-Fornier B. Regulation of amino acid, nucleotide, and phosphate metabolism in Saccharomyces cerevisiae. Genetics. 2012; 190:885–929. 22419079 10.1534/genetics.111.133306 PMC3296254

[48] Satyanarayana T., Kaplan J.G. Regulation of the purine pathway in bakers yeast: activity and feedback inhibition of phosphoribosyl-pyrophosphate amidotransferase. Arch. Biochem. Biophys. 1971; 142:40–47. 4322810 10.1016/0003-9861(71)90257-8

[49] McWilliam H., Li W., Uludag M., Squizzato S., Park Y.M., Buso N., Cowley A.P., Lopez R. Analysis tool web services from the EMBL-EBI. Nucleic Acids Res. 2013; 41:W597–W600. 23671338 10.1093/nar/gkt376 PMC3692137

[50] Larkin M.A., Blackshields G., Brown N.P., Chenna R., McGettigan P.A., McWilliam H., Valentin F., Wallace I.M., Wilm A., Lopez R. et al. Clustal W and Clustal X version 2.0. Bioinformatics. 2007; 23:2947–2948. 17846036 10.1093/bioinformatics/btm404

