# Supplementary material for: Phylogenetic debugging of a complete human biosynthetic pathway transplanted into yeast
Source: Nucleic Acids Res. 2019 Nov 20;48(1):486–99. doi: 10.1093/nar/gkz1098 (PMC7145547; doi:10.1093/nar/gkz1098)
Supplement: gkz1098_Supplemental_Files [file gkz1098_supplemental_files.zip › Supplementary Figures_NAR_Final.pdf]

Supplementary Figures:

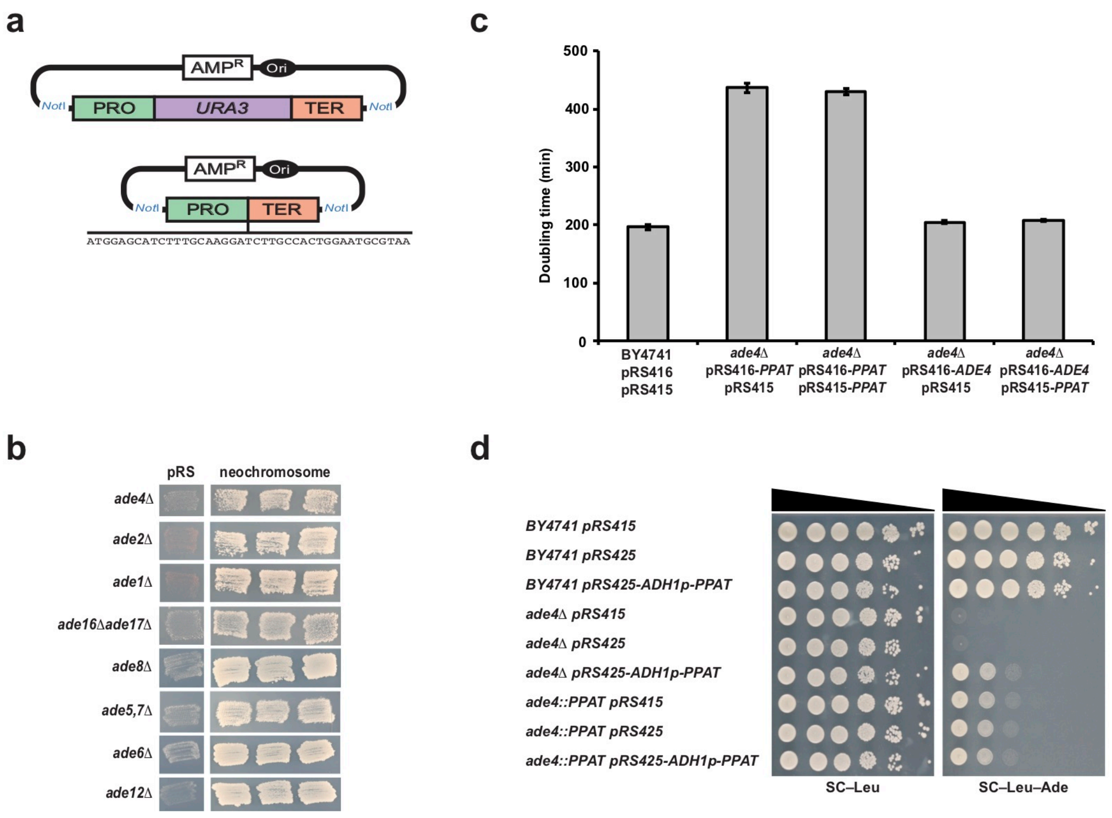

Supplementary Figure 1: humanized yeast assembly and examination and PPAT copy number increase. (a) In order to delete the yeast *ADE* genes, we used yGG to assemble two constructs for each gene: a *URA3* integrating cassette (left) flanked with the gene's promoter and terminator and a Linker cassette (right) also flanked with the gene's promoter and terminator. **(b)** Single gene complementation by the adenine de novo neochromosome. Three individual colonies from the transformation were assayed for their ability to grow on media without adenine. For *ade16* and *ade17*, adenine auxotrophy is only observed in a double mutant. **(c)** Graphic representation of doubling time in medium without adenine of wild-type (WT; BY4741) and *ade4*Δ strains carrying *PPAT* on plasmid with different copy number and under different promoters. Doubling time was high with both one or two copies of the PPAT gene, only in those strains carrying *ADE4* plasmid was the doubling time similar to wild type. **(d)** A dot assay examining the effect of overexpression of *PPAT*. Increasing expression of *PPAT* did not have an effect of growth without adenine. Doubling time was calculated from growth curves with mean and standard deviations from 8 replicates.

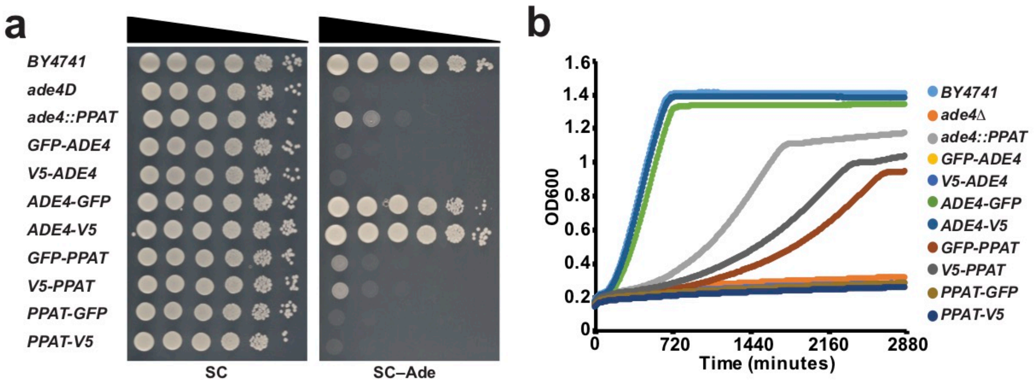

Supplementary Figure 2: Protein tagging of Ade4 and Ppat and its effect on growth without adenine. (a) A dot assay examining the effect of C-terminal and N-terminal tagging of Ade4 and Ppat on growth on media without adenine. **(b)** Graphic representation examining the effect of C-terminal and N-terminal tagging of Ade4 and Ppat on growth rate in media without adenine. Both assays show that Ade4 could only be tagged on the C-terminus and Ppat could only be tagged on the N-terminus whilst conserving enzymatic activity. V5 tag shows slightly better growth versus GFP tag.

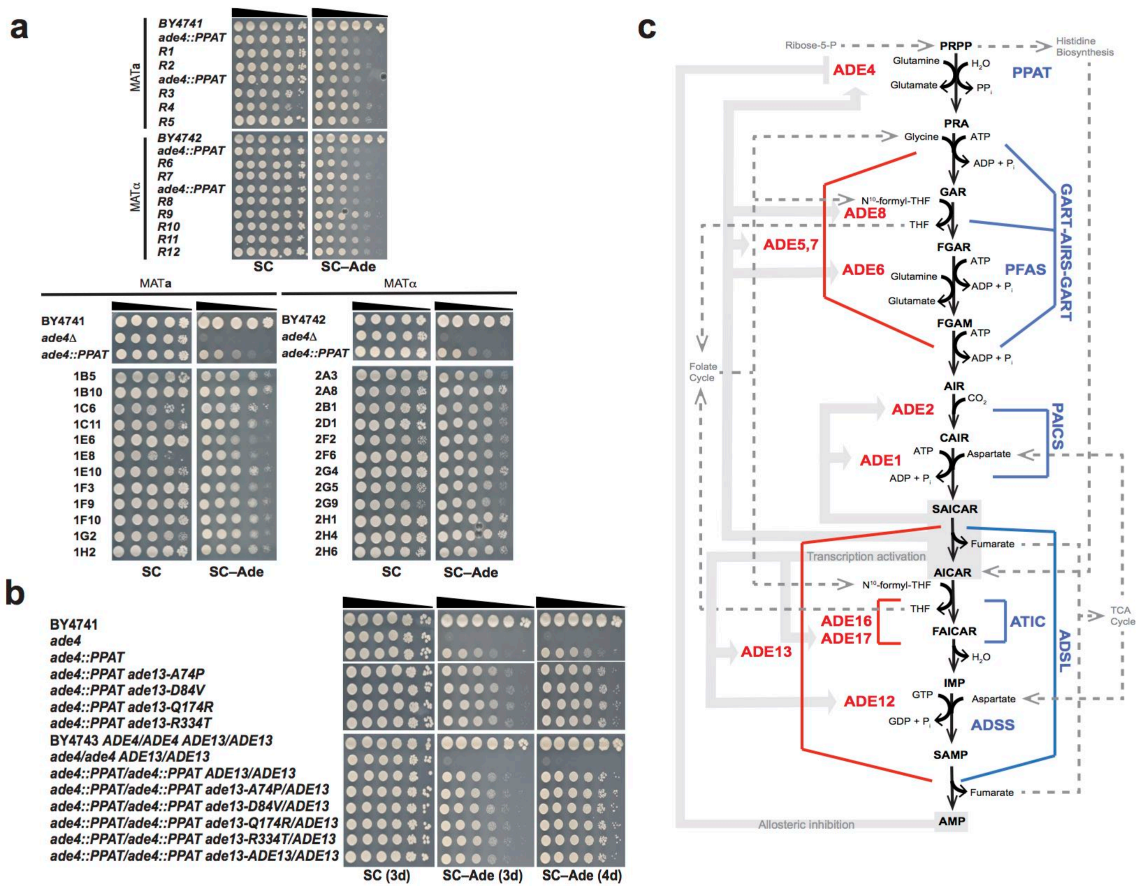

Supplementary Figure 3: Suppressor analysis and adenine de novo pathway regulation. (a) Dot assays showing growth of *ade4::PPAT* suppressors with and without adenine. All suppressor strains show increased growth without adenine. All 36 suppressor strains were sequenced to identify mutations affecting the phenotype (Supplementary Table S1). **(b)** Dot assays showing the growth of haploids *PPAT* strains carrying *ade13* suppressor mutation versus diploid *PPAT* homozygous strains that are heterozygous for *ade13* suppressor mutations. Growth on media without adenine shows that *ade13* mutation alleviates the growth defect of the *PPAT* strain (compare *ade4::PPAT* to *ade4::PPAT* ade13-X), while in diploid strains comparing the growth on *PPAT* (*ade4::PPAT/ade4::PPAT ADE13/ADE13*) to the strains heterozygous to *ade13* suppressor mutations (*ade4::PPAT/ade4::PPAT ade13-X/ADE13*) there is no difference in growth. Thus, indicates that the mutations are recessive and thus most likely hypomorphs. **(c)** Schematic representation of the adenine de-novo biosynthesis pathway. Red, yeast genes; blue human. Grey dotted lines represent different metabolic pathways that interact with the adenine de-novo pathway through its metabolites. Background grey arrows indicate previously known transcriptional and allosteric regulation.

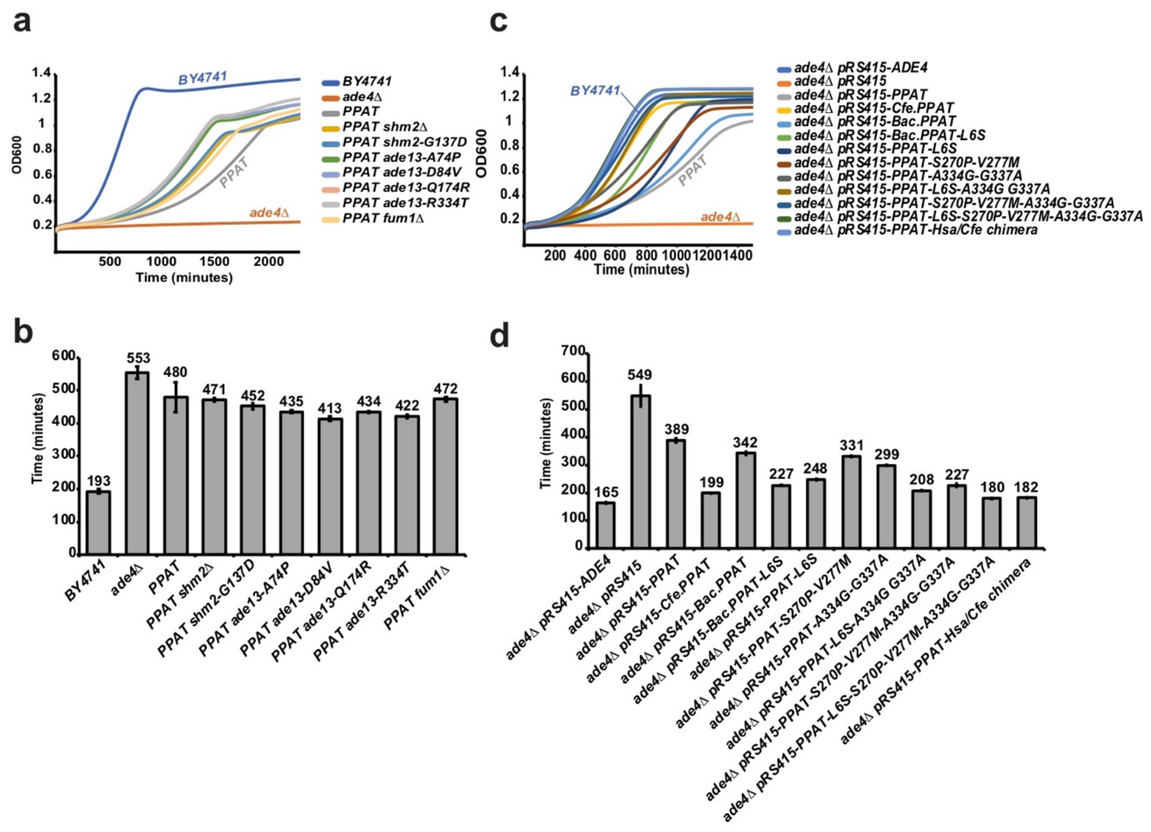

Supplementary Figure 4: Improvement in growth in media without adenine in PPAT cells. (a) Graphic representation examining the effect of the different suppressors on PPAT cells grown in media without adenine. Similar to the results shown in Fig. 2A, all suppressors show a mild rescue of the growth rate compared to PPAT alone. **(b)** Graphic representation of doubling time in medium without adenine of wild-type (WT; BY4741) and *ade4*Δ and *PPAT* strains as well as *PPAT* strains carrying the different suppressors. All suppressors show a mild effect on doubling time compared to *PPAT* strains. **(c)** Graphic representation of growth curves of *ade4Δ* strains carrying low copy number plasmids cloned with *ADE4*, *PPAT* (*Hsa.PPAT*), Camel *PPAT* (*Cfe.PPAT*), Whale *PPAT* (*Bac.PPAT*) and the different mutation found in our DNA shuffle experiment. **(d)** Graphic representation of doubling time in medium without adenine of *ade4Δ* strains carrying low copy number plasmids cloned with *ADE4*, *PPAT* (*Hsa.PPAT*), Camel *PPAT* (*Cfe.PPAT*), Whale *PPAT* (*Bac.PPAT*) and the different mutation found in our DNA shuffle experiment. Different combinations of the 5 mutations show different effect on the doubling time, only *Cfe.PPAT*, Has/Cfe chimera *PPAT* and the human *PPAT* carrying all 5 mutations show growth closest to *ADE4* carrying strains.

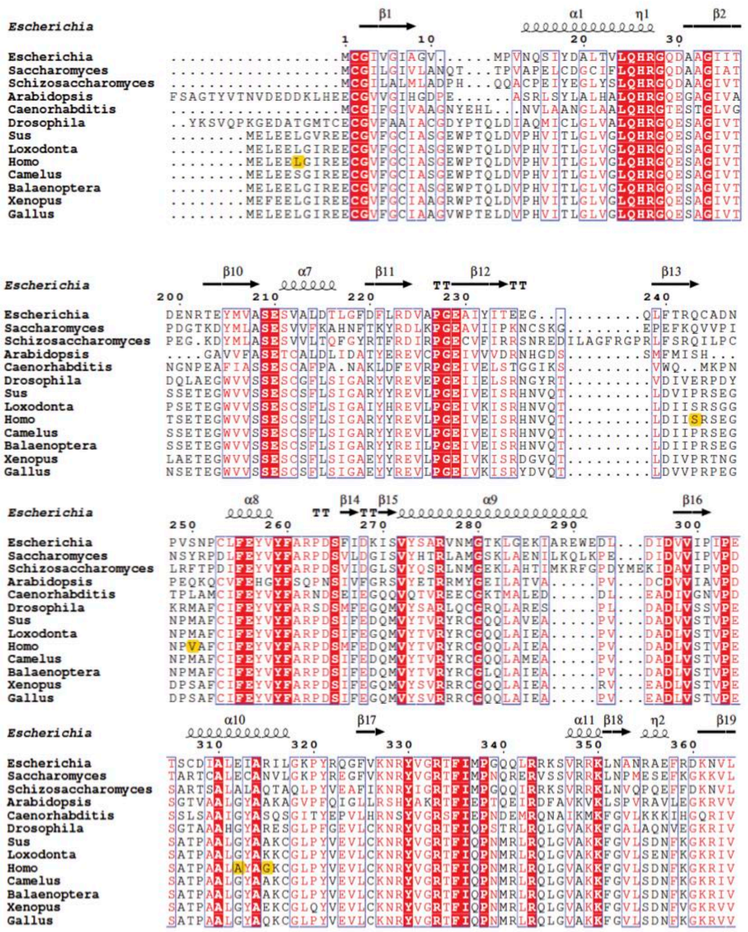

Supplementary Figure 5: Multiple sequence alignment of PPAT protein. Multiple protein sequence alignment to *E. coli purF* secondary structure elements (PDB DOI 1 ECB) of the protein N-terminal domain (upper panel) and of the PRPP binding domain (Three lower panels). Multiple sequence alignment was performed using CLUSTAL-OMEGA(43) , alignment to protein structure was performed using the ESPript 3.0 web server(51). Identical residues are boxed in red. The 5 residues substituted in highly active variants produced by DNA shuffling experiments compared to the human PPAT, are marked in yellow on the human sequence.

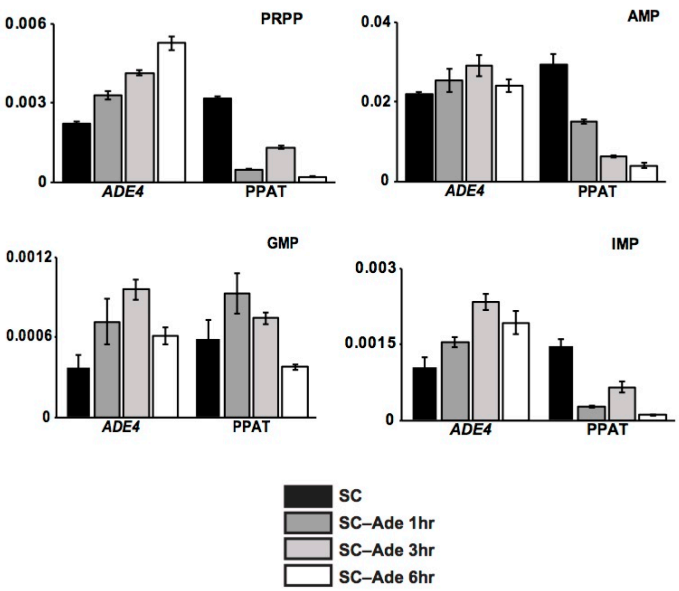

Supplementary Figure 6: Metabolite analysis preformed on prototrophic WT cells vs. prototrophic cells expressing PPAT. PPAT is integrated into *ADE4* locus and expresses from its native promoter. PRPP, AMP, GMP and IMP levels are shown in SC (black), SC–Ade for 1h. (dark grey), 3 h. (light grey) and 6 h. (white). Metabolomics raw data is presented in Supplementary Table S3.

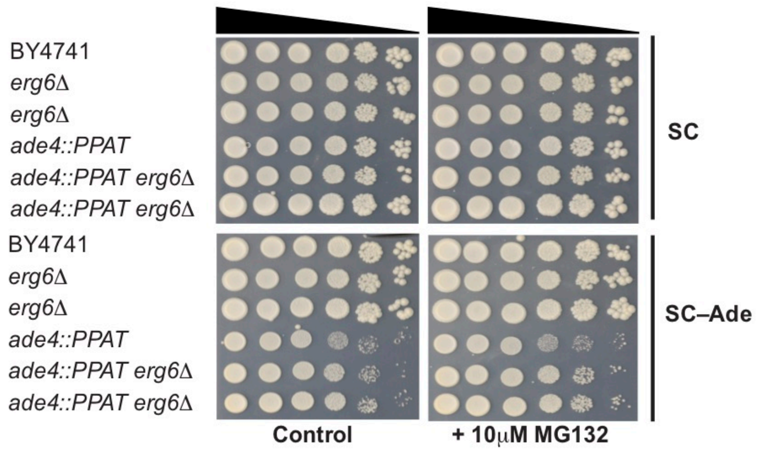

Supplementary Figure 7: Inhibition of the proteasome partially rescues PPAT growth rate on media without adenine. A dot assay examining the effect of proteasome inhibitor, MG132, on *PPAT* complementation in *ade4Δ* cells. PPAT cells deleted for *erg6* on media supplemented with MG132 complement better than *ERG5 PPAT* cells.
